# Supplementary material for: Electron penetration triggering interface activity of Pt-graphene for CO oxidation at room temperature
Source: Nat Commun. 2021 Oct 4;12:5814. doi: 10.1038/s41467-021-26089-y (PMC8490350; doi:10.1038/s41467-021-26089-y)
Supplement: Supplementary file 1 — Supplementary Information [file 41467_2021_26089_MOESM1_ESM.pdf]

Supporting Information

Electron Penetration Triggering Interface Activity of Pt-Graphene for CO Oxidation at Room Temperature

Yong Wang[1,2,7], Pengju Ren[3,4,7], Jingting Hu[1,2], Yunchuan Tu[1,2], Zhongmiao Gong[5], Yi Cui[5], Yanping Zheng[1], Mingshu Chen[1], Wujun Zhang[6], Chao Ma[6], Liang Yu[2], Fan Yang[2], Ye Wang[1], Xinhe Bao[2], Dehui Deng[1,2]*

[1]State Key Laboratory of Physical Chemistry of Solid Surfaces, Collaborative Innovation Center of Chemistry for Energy Materials (*i*ChEM), College of Chemistry and Chemical Engineering, Xiamen University, 361005 Xiamen, China

[2]State Key Laboratory of Catalysis, *i*ChEM, Dalian Institute of Chemical Physics, Chinese Academy of Sciences, 116023 Dalian, China

[3]State Key Laboratory of Coal Conversion, Institute of Coal Chemistry, Chinese Academy of Sciences, 030001 Taiyuan, China

[4]National Energy Center for Coal to Liquids, Synfuels China Co. Ltd., 101407 Beijing, China

[5]Vacuum Interconnected Nanotech Workstation, Suzhou Institute of Nano-Tech and Nano-Bionics, Chinese Academy of Sciences, 215123 Suzhou, China

[6]Center for High Resolution Electron Microscopy, College of Materials Science and Engineering, Hunan University, 410082 Changsha, China

[7]These authors contributed equally: Yong Wang, Pengju Ren

*Corresponding author. Email: dhdeng@dicp.ac.cn

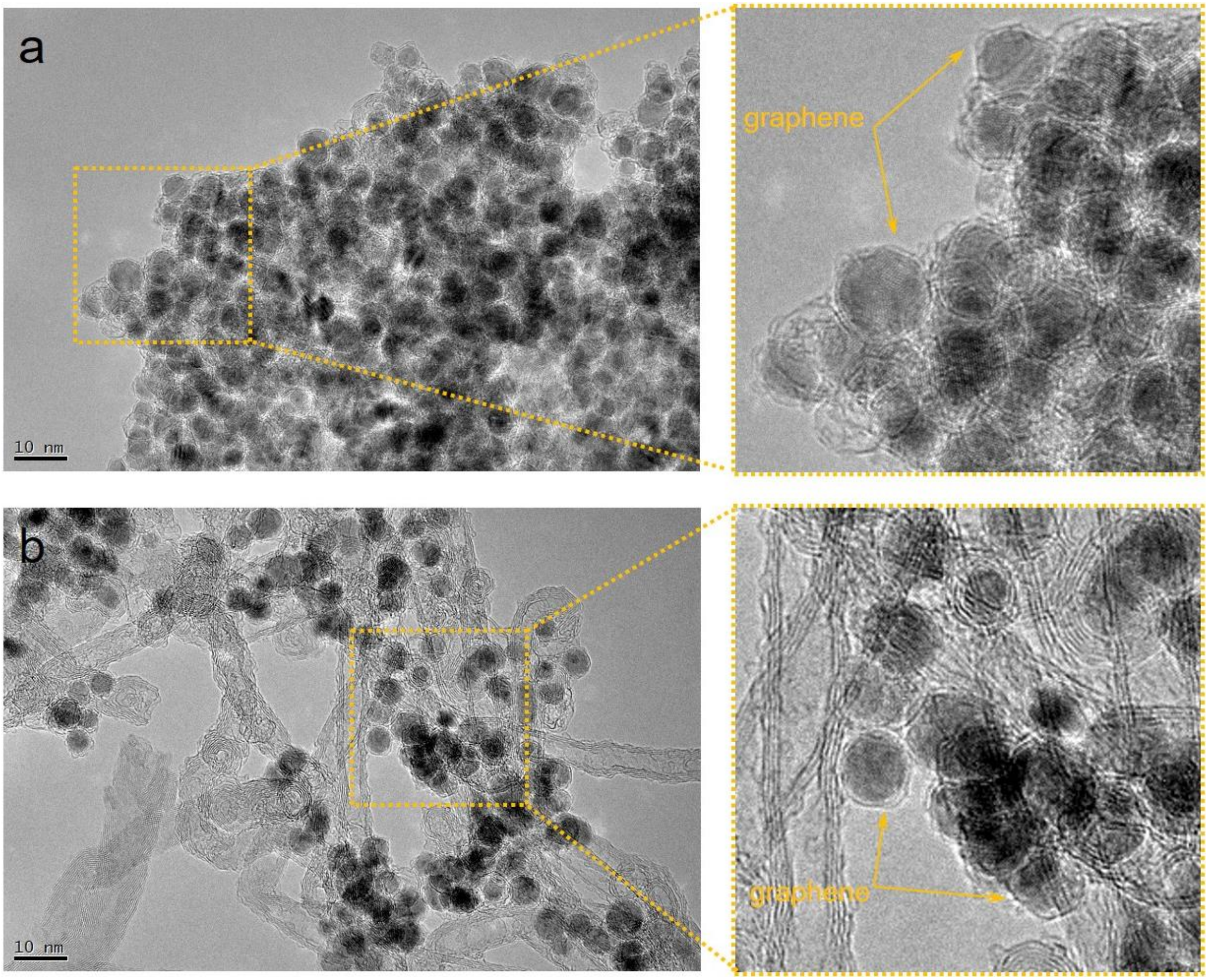

Supplementary Fig. 1 TEM images of (a) CoNi@C and (b) CoNi@NC**.**

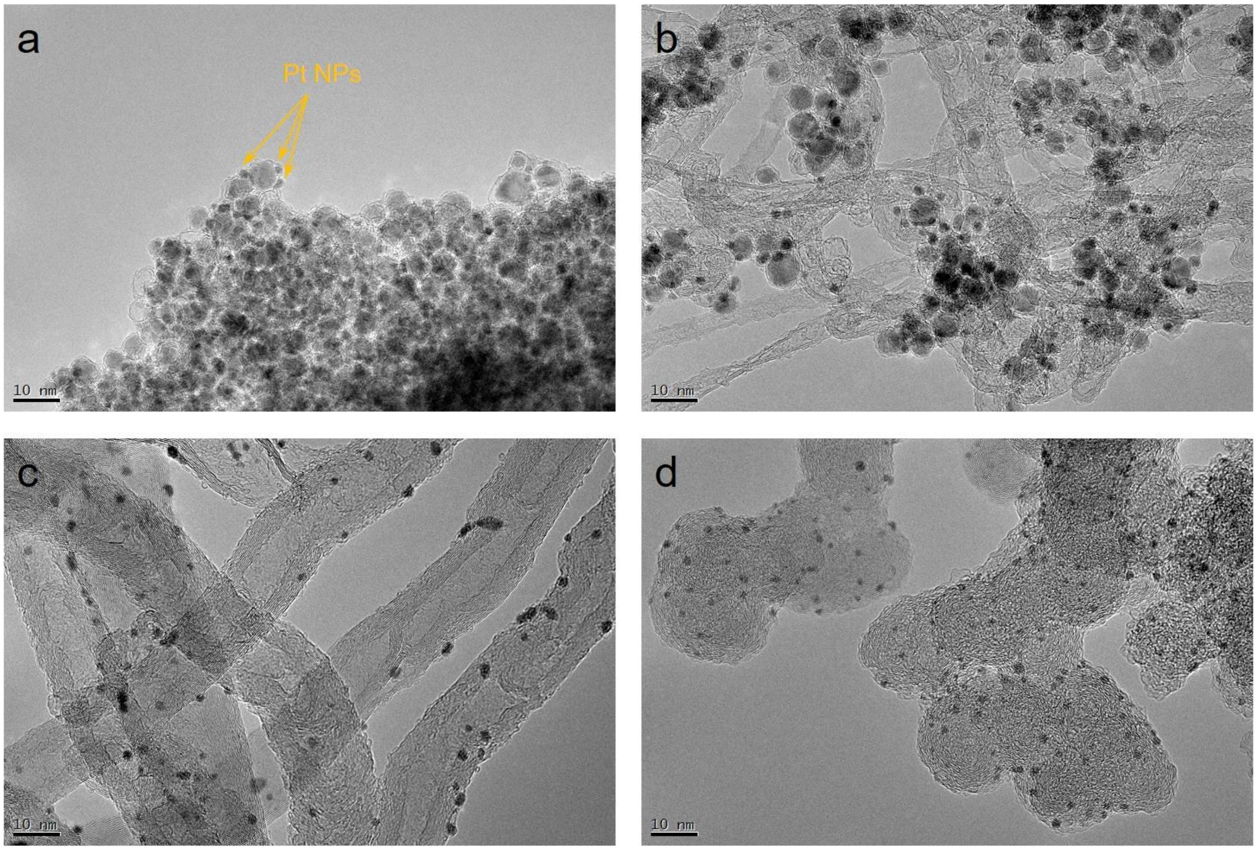

Supplementary Fig. 2 TEM images of (a) Pt/CoNi@C, (b) Pt/CoNi@NC, (c) Pt/CNT, and (d) Pt/CB.

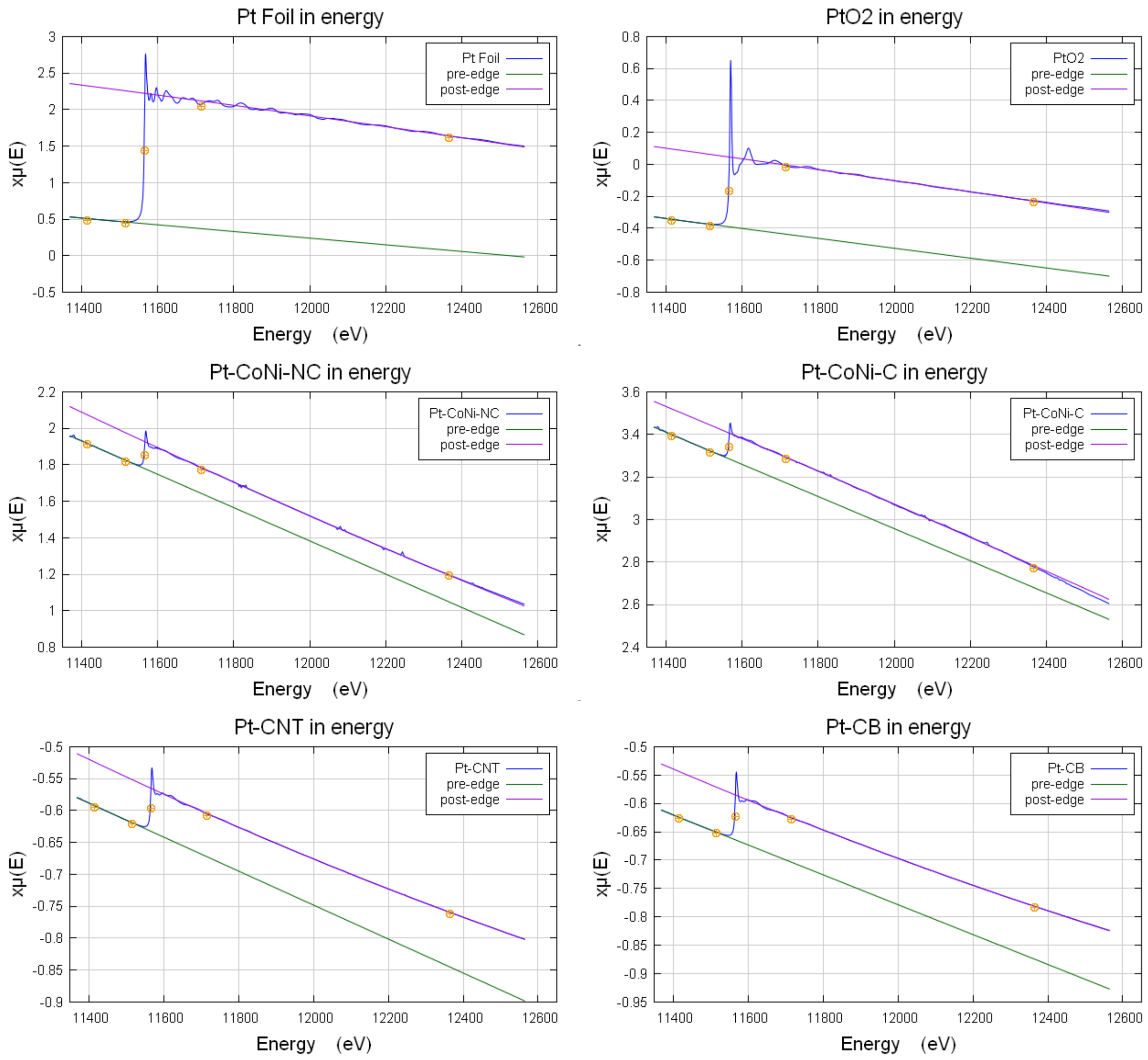

Supplementary Fig. 3 XAS spectra of Pt foil, PtO_2, Pt/CoNi@NC, Pt/CoNi@C, Pt/CNT, and Pt/CB with pre-edge (–180 to –80 eV relative to E_0) and post-edge (+150 to +800 eV relative to E_0) baselines before normalization. The E_0 of all spectra is calibrated to 11564.0 eV with the same energy shift of –0.1 eV. The absorption strength is weaker for the four catalysts samples than Pt foil and PtO_2 due to their low Pt loadings. The spectrum of Pt/CoNi@C has a slight bend at the high energy region probably due to the background drift.

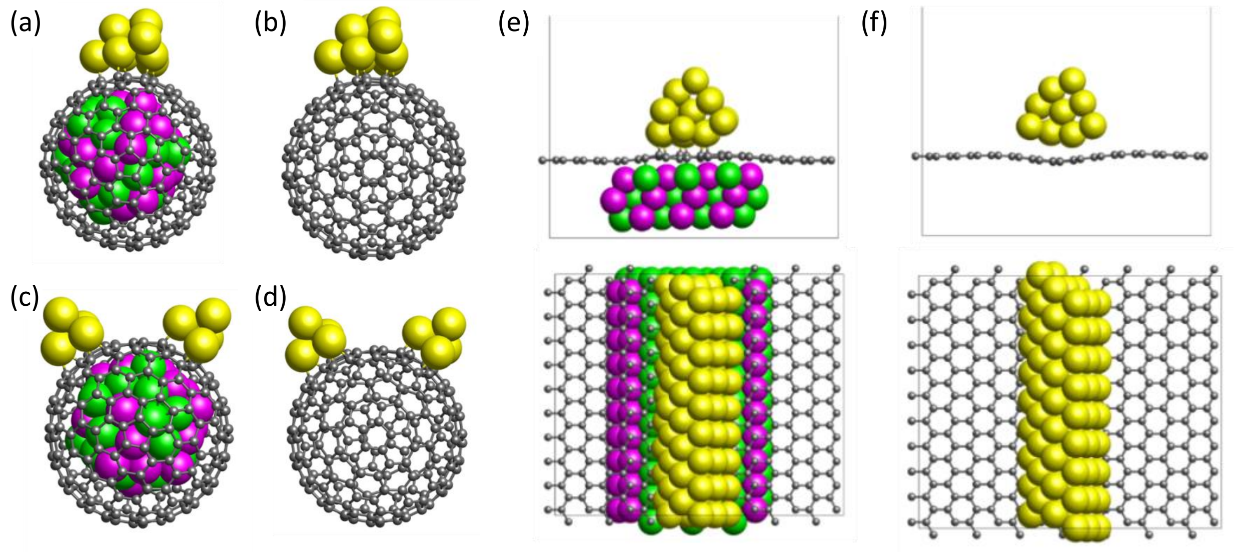

Supplementary Fig. 4 Comparable models of (a,b) Pt_9 cluster on graphene cage, (c,d) two Pt_4 clusters on graphene cage, and (e,f) Pt nano-strip on graphene layer. The grey, yellow, green, and pink balls represent C, Pt, Co, and Ni, respectively. The corresponding Barder charge of Pt cluster and the binding energy between Pt and C are summarized in Table S1, from where we can observe the same rule of electron transfer and strengthened binding energy due to the electron penetration effect.

Supplementary Table 1. Barder charge of Pt cluster and binding energy between Pt and C on different models.

Model	Pt_4		Pt_9		$2Pt_4$		Pt nano-strip	
	CoNi@C	C	CoNi@C	C	CoNi@C	C	CoNi@C	C
Charge	–0.08	+0.15	–0.09	+0.13	–0.12	+0.26	–0.32	+0.80
E_b	–2.74	–2.17	–2.74	–1.38	–5.50	–4.24	–28.66	–18.85

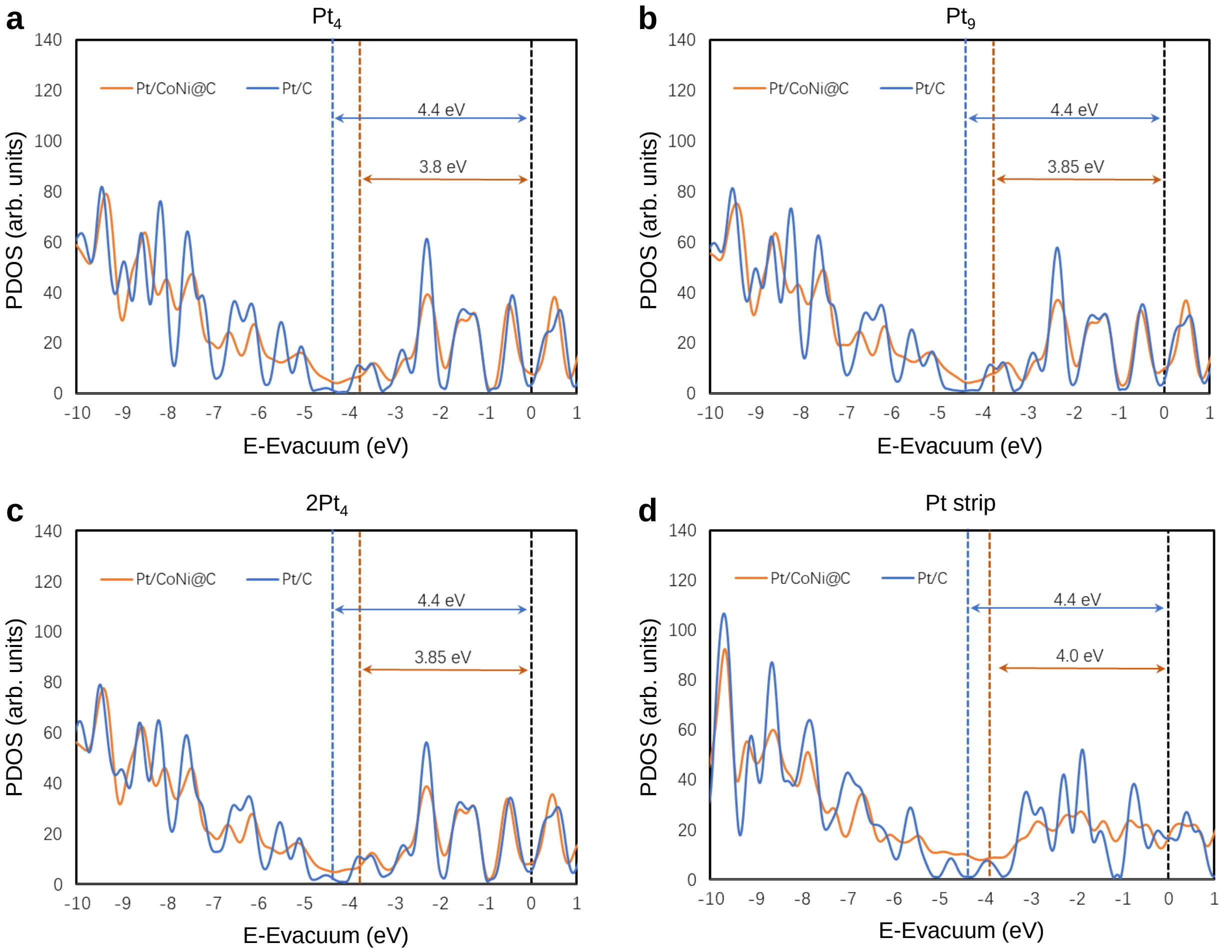

Supplementary Fig. 5 Comparison of PDOS of 2*s*+2*p* orbitals of C atoms between (a) Pt_4/CoNi@C and Pt_4/C, (b) Pt_9/CoNi@C and Pt_9/C, (c) $2Pt_4$/CoNi@C and $2Pt_4$/C, and (d) Pt_{strip}/CoNi@C and Pt_{strip}/C.

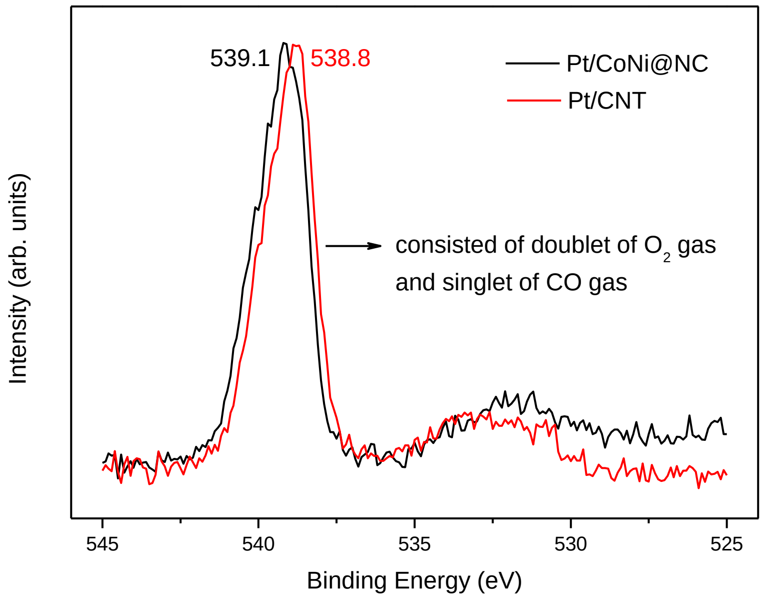

Supplementary Fig. 6 XPS spectra of O 1*s* from NAP-XPS testings over Pt/CoNi@NC and Pt/CNT in a flow of 0.067 mbar CO and 1.13 mbar O_2 at room temperature. The peak at around 539 eV is attributed to the signals form CO and O_2 gas. The doublet of O_2 merged together, and the CO signal may also merge into the whole peak if it can be detected. However, we can still observe that this peak of gas phase over Pt/CoNi@NC has a clear blue shift of 0.3 eV relative to that over Pt/CNT. As proposed by Liu et al. (Ref. 22), there is a negative correlation between the work function of material surface and the binding energy of gas phase over it. Thus, this data experimentally proved the decrease of work function of graphene in Pt/CoNi@NC.

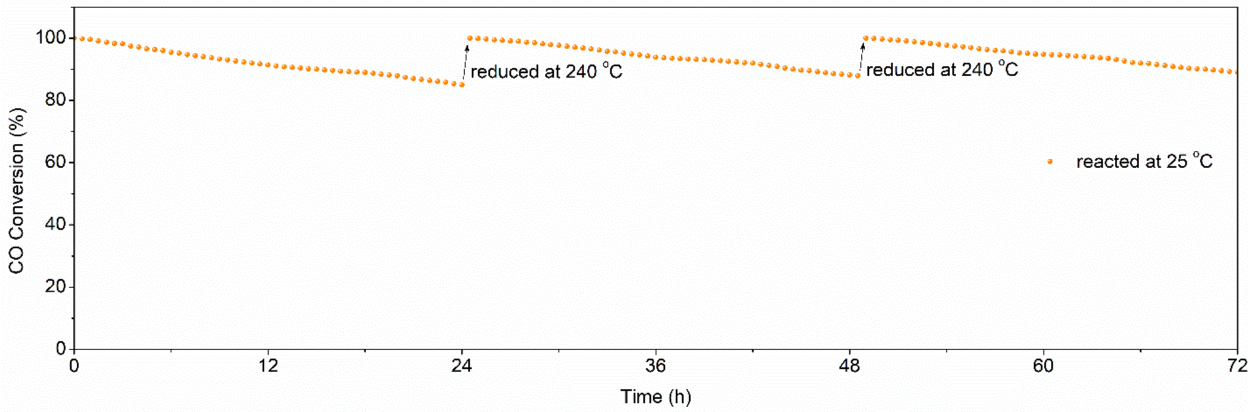

Supplementary Fig. 7 Long-term stability of Pt/CoNi@NC for the CO oxidation at 25 °C under a mixed flow of 1% CO and 20% O_2 in He (1 bar) with a total space velocity of 60000 mL·g^{-1}·h^{-1}. After each 24 h, catalyst was reduced in H_2 at 240 °C for 2 h. The slow deactivation may be due to either the gradual CO poison on Pt NPs or the possible oxidation of Pt NPs, which is still under investigation.

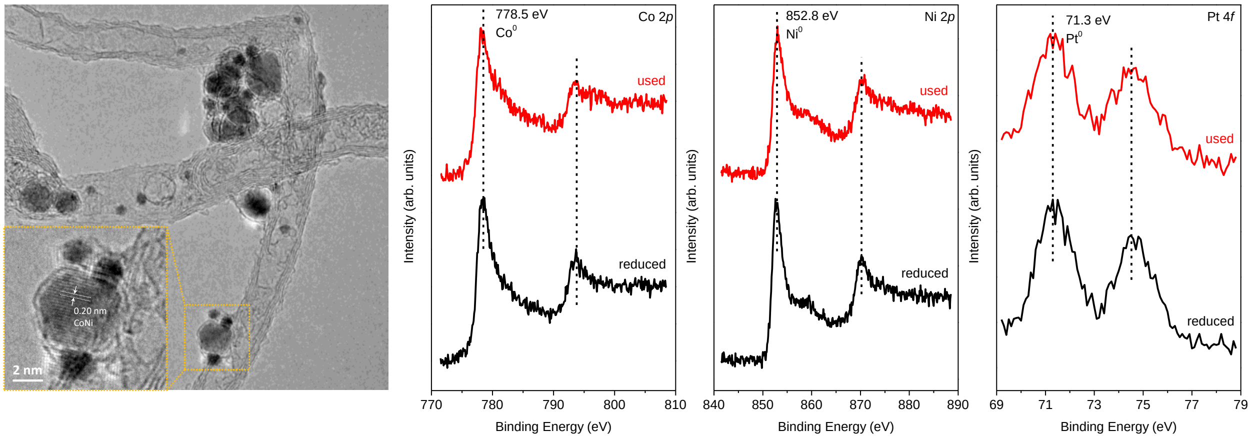

Supplementary Fig. 8 TEM image and XPS spectra of Pt/CoNi@NC after reacting at 25 °C for 24 h.

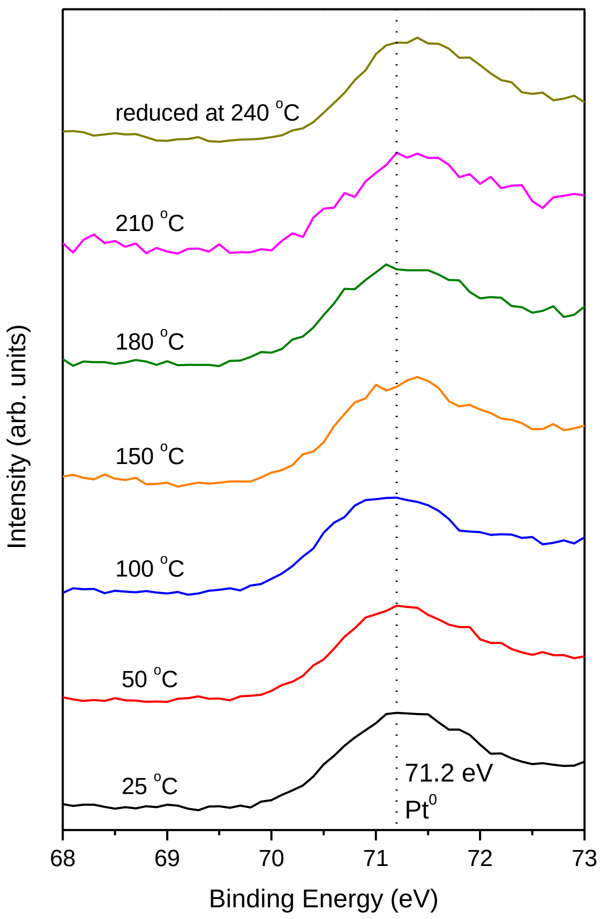

Supplementary Fig. 9 XPS spectra of Pt 4$f_{7/2}$ from NAP-XPS testings over Pt/CoNi@NC in a flow of 0.067 mbar CO and 1.13 mbar O_2 at different reaction temperatures (25-210 °C). The reduction process was performed after reaction.

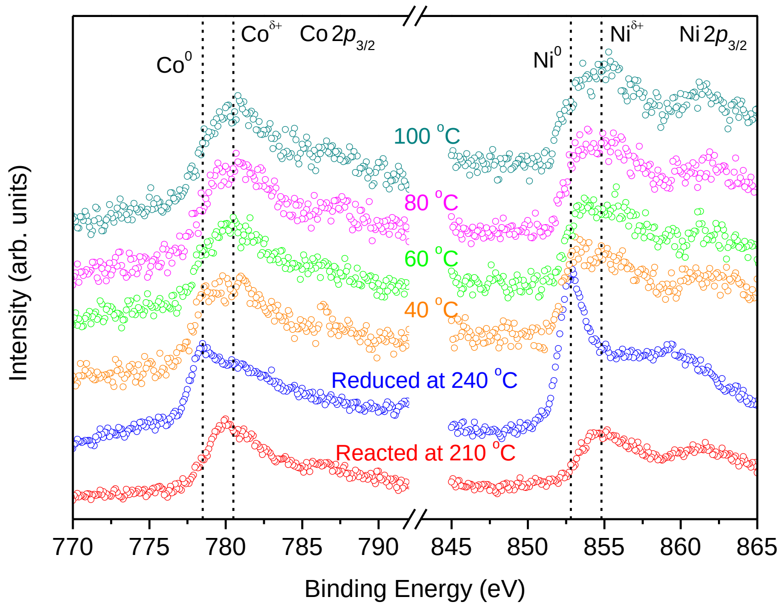

Supplementary Fig. 10 Co 2$p_{3/2}$ and Ni 2$p_{3/2}$ spectra during the NAP-XPS testings over Pt/CoNi@NC. After the in-situ NAP-XPS testings shown in Fig. 2b, the inner CoNi NPs were oxidized due to the oxidative destruction of graphene. Then, the catalyst was re-reduced at 240 °C for 1 h (the bule circles in Supplementary Fig. 10). After cooling to around 40 °C under vacuum, a mixed gas of 0.067 mbar CO and 1.13 mbar O_2 flowed through the catalyst for reaction from 40 to 100 °C. One can see that once exposed to the mixed gas, the reduced CoNi can be rapidly oxidized even at 40 °C without the protection of graphene.

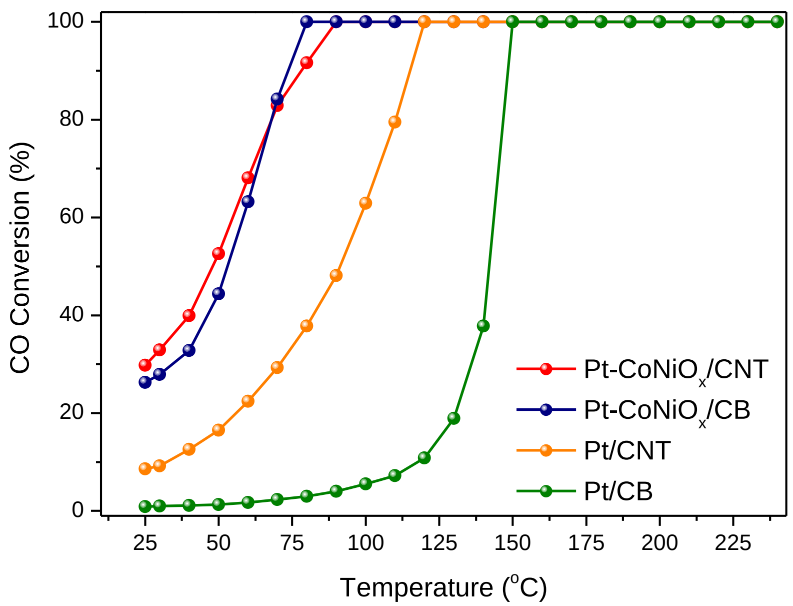

Supplementary Fig. 11 Temperature-dependence CO conversion in CO oxidation reaction over the pre-reduced catalysts. 1% CO and 20% O_2 in He (1 bar). Space velocity: 60000 $mL{\cdot}g^{-1}{\cdot}h^{-1}$. Pt-$CoNiO_x$/CB and Pt-$CoNiO_x$/CNT catalysts were prepared as follows. First, $CoNiO_x$ was precipitated on carbon supports in a form of layered double hydroxides with metals loading of 10 wt%. The procedure is the same as that for $CoNiO_x/SiO_2$ described in the section of Methods. Then, Pt NPs (1-2 nm) were deposited on $CoNiO_x$/CB and $CoNiO_x$/CNT with a Pt loading of 4 wt%. The procedure is the same as that for Pt/CoNi@NC described in the section of Methods. From Supplementary Fig. 11, one can see that the catalytic activity is enhanced after introducing $CoNiO_x$ onto both carbon supports and quite close for Pt-$CoNiO_x$/CB and Pt-$CoNiO_x$/CNT. The minor support effect indicated that most of Pt NPs should be in close contact with $CoNiO_x$ on both catalysts. The formed Pt-$CoNiO_x$ interfaces presented a much lower activity of CO oxidation than the fresh Pt/CoNi@NC catalyst (Fig. 2a). It implies that the activity would decrease if Co or Ni escapes from the graphene shell during reaction, and this has been confirmed by the experimental design shown in Fig. 2d. However, the activity of the broken Pt/CoNi@NC catalyst (Fig. 2d) is a little lower than that of Pt-$CoNiO_x$/CNT, which may be due to a lower number of Pt-$CoNiO_x$ interfaces for the former, because there are still some Pt NPs deposited on the concomitant CNT in Pt/CoNi@NC.

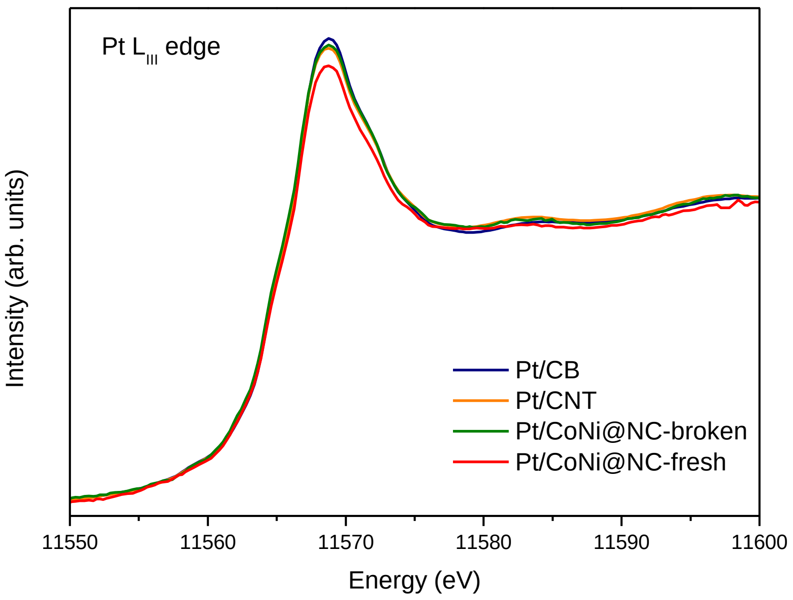

Supplementary Fig. 12 XANES spectra of Pt L_{III} edge for Pt/CB, Pt/CNT, fresh and broken Pt/CoNi@NC. The broken Pt/CoNi@NC is the sample after reacting to 240 °C (after the 2nd run in Fig. 2d). The jump height of Pt in the broken Pt/CoNi@NC is higher than that in the fresh sample, indicating a decreased *d*-band electron density.

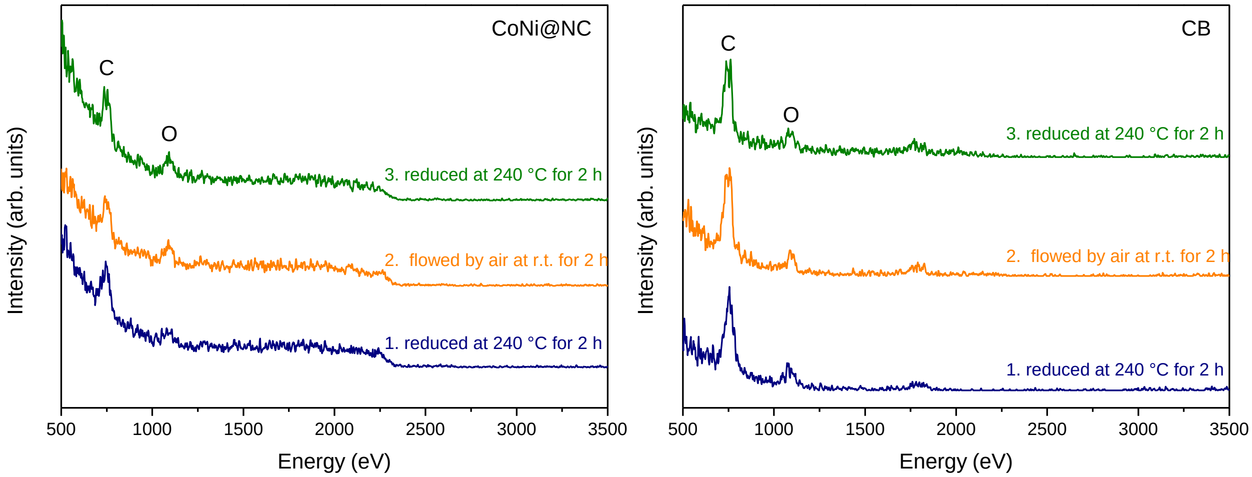

Supplementary Fig. 13 LEIS spectra of CoNi@NC and CB after exposing in air and reduction in H_2. The O signal of CoNi@NC increased after exposing in air flow at room temperature for 2 h and cannot be efficiently removed by reduction, which is consistent with the results of Pt/CoNi@NC shown in Fig. 2e. In contrast, the O signal of CB did not change two much after these treatments. Thus, O_2 can be activated on CoNi@NC but not on CB at room temperature, which can further confirm that the graphene turns to be more active when encapsulating CoNi NPs (Supplementary Fig. 5).

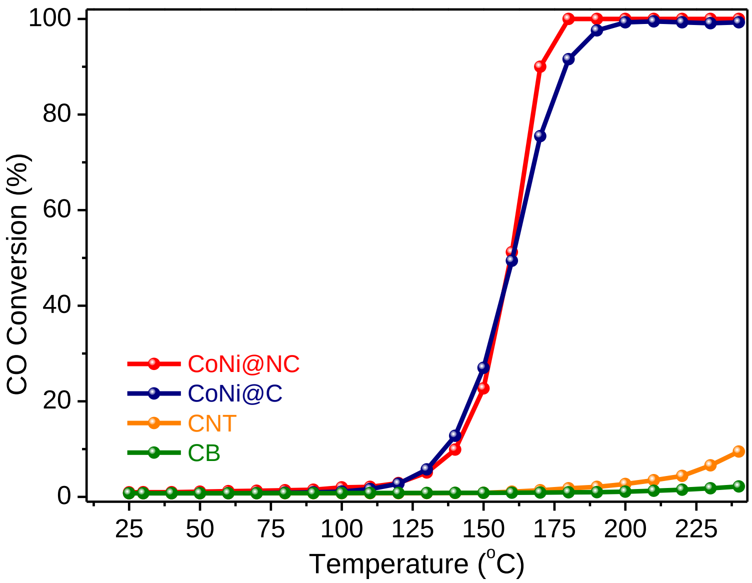

Supplementary Fig. 14 Temperature-dependence CO conversion in CO oxidation reaction over the pre-reduced supports under a mixed flow of 1% CO and 20% O_2 in He (1 bar) with a space velocity of 60000 $mL \cdot g^{-1} \cdot h^{-1}$. This chart shows the activities of four supports without loading Pt. Graphene encapsulating CoNi alloy cannot catalyze the reaction until 120 °C. In comparison with the room-temperature activity of catalysts with Pt (Fig. 2a), one can see that Pt is essential for the activation of CO considering that O_2 can be activated on the graphene surface of graphene encapsulating CoNi alloy at room temperature (Supplementary Fig. 13).

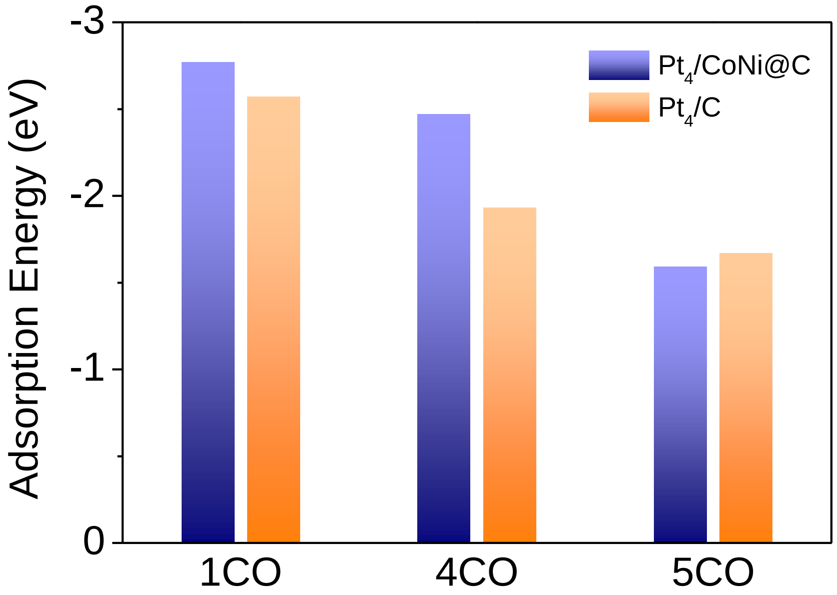

Supplementary Fig. 15 Adsorption energy of CO on Pt_4/CoNi@C and Pt_4/C with different coverages. Here, the adsorption energy of 4CO and 5CO refers to the energy of the 4th and 5th CO adsorbing on the 3CO-adsorbed and 4CO-adsorbed Pt_4 clusters, respectively. 5CO can reach the saturated coverage on our Pt_4 cluster. It can be seen that the adsorption of CO on both Pt_4/CoNi@C and Pt_4/C will be weakened along with increasing the coverage of CO. The same rule applies for the adsorption of O_2. For example, the adsorption energy of O_2 at the Pt-graphene interfaces of Pt_4/CoNi@C decreases from –0.37 to –0.21 eV when saturating with 5CO on Pt_4 cluster. However, the adsorption of the 5th CO on 4CO-adsorbed Pt_4 (–1.6 eV) is still stronger than that of O_2 on a clean Pt_4 (–1 eV, Fig. 3a), indicating that O_2 cannot compete with CO for adsorption on Pt site no matter with the coverage of CO. Thus, we start the energy profile form 4CO-adsorbed Pt_4 (model I in Fig. 3b), and the first step is the adsorption of the 5th CO on it to reach the saturated coverage.

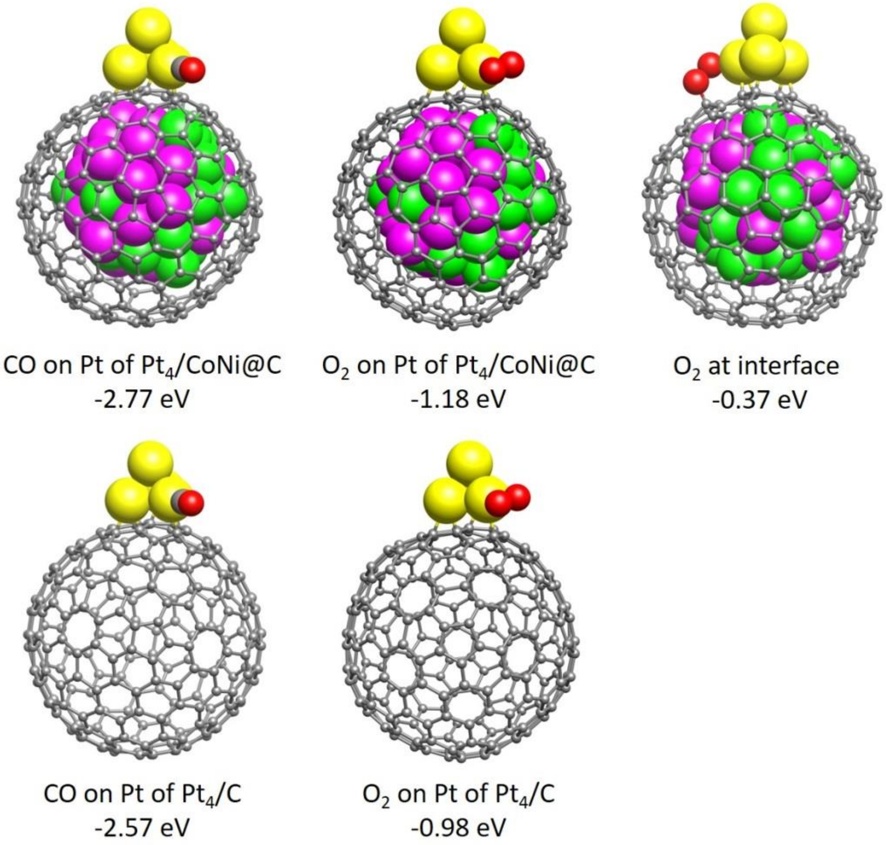

Supplementary Fig. 16 Adsorption models and energies of CO and O_2 on Pt_4/CoNi@C and Pt_4/C. The red, grey, yellow, green, and pink balls represent O, C, Pt, Co, and Ni, respectively.

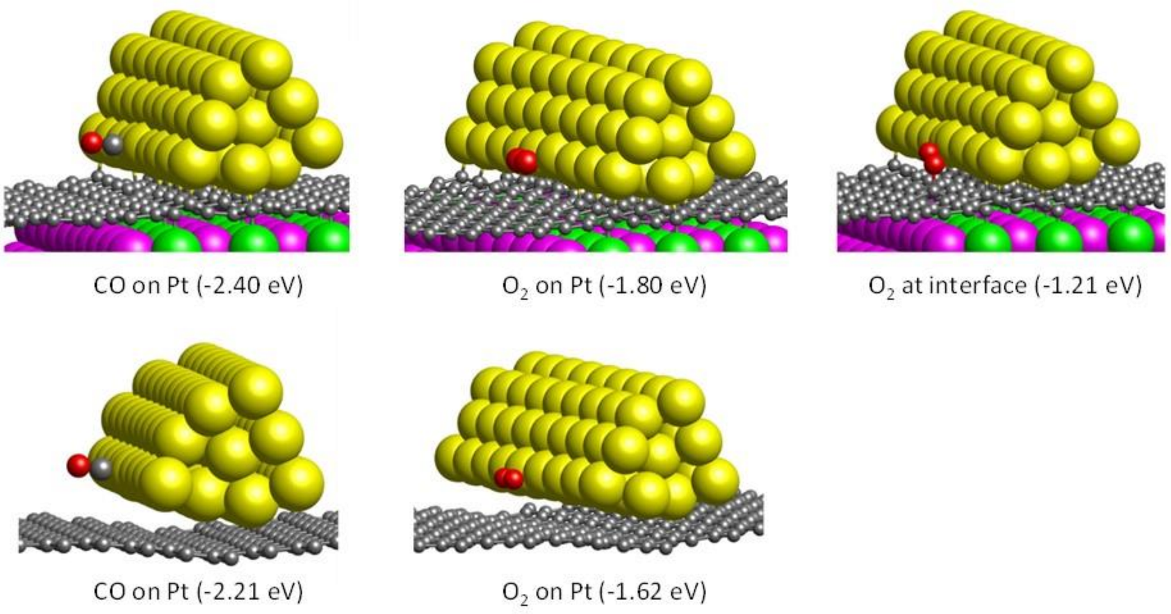

Supplementary Fig. 17 Adsorption models and energies of CO and O_2 on Pt_{strip}/graphene/CoNi and Pt_{strip}/graphene. The red, grey, yellow, green, and pink balls represent O, C, Pt, Co, and Ni, respectively.
